# Watching the South China Sea—*Portiodora* (*Iridaceae*), a New Genus for *Iris speculatrix* Based on Comprehensive Evidence: The Contribution of Taxonomic Resolution to Biodiversity Conservation

**DOI:** 10.3390/biology14121767

**Published:** 2025-12-11

**Authors:** Manuel B. Crespo, Mario Martínez-Azorín, Evgeny V. Mavrodiev

**Affiliations:** 1 Departamento de Ciencias Ambientales y Recursos Naturales (dCARN), Universidad de Alicante, P.O. Box 99, ES-03080 Alicante, Spain; mmartinez@ua.es; 2 Department of Botany, Rhodes University, Makhanda 6140, South Africa; 3 Florida Museum of Natural History, University of Florida, Gainesville, FL 32611, USA; evgeny@ufl.edu

**Keywords:** Asparagales, *Iris* subg. *Limniris*, new genus, new combinations, nomenclature, integrative taxonomy, phylogenetics, plant conservation, eastern Asia

## Abstract

**Simple Summary:**

The Hong Kong iris (*Iris speculatrix*) is an enigmatic species native to southeastern China that is under threat of extinction. By comparing morphological and molecular data, we found that it truly occupies a unique position within the “*Iris*-flower clade” and is indeed a sister species to a wider clade of beardless and uncrested irises with much broader distributions. A re-evaluation of the ecology, karyology, distribution and phylogenetic relationships of *I. speculatrix* was conducted, which resulted in the establishment of the new genus *Portiodora* for accurate classification of the species. We confirmed that the pure comparative approach to molecular data (three-taxon statement analysis), which excludes assumptions about the history of chloroplast loci transformation, is practical for addressing taxonomic questions and may therefore be applicable to similar studies. We have provided an identification key for the entire “*Iris*-flower clade” to frame the unique morphology of *Portiodora* in the context of the whole clade. A deep understanding of the uniqueness of *P. speculatrix* is crucial for plant conservation efforts in southeastern China. However, our study provides a framework for a future protective strategy for the entire “*Iris*-flower clade”, since accurately resolving the taxonomy of cryptic biodiversity may be essential for its conservation.

**Abstract:**

*Iris speculatrix* was described from plants collected in Hong Kong and is accepted to extend through southeastern China. The species is scheduled under the Forestry Regulations and is regarded as endangered (EN) according to the IUCN categories. This enigmatic plant exhibits multiple morphological connections to other congeners, and was classified in contrasting groups such as the “Chinenses” or the “Ensatae” irises. Molecular work placed it as an isolated lineage sister to the beardless/uncrested subgroup of the so-called “*Xiphion* s.l. clade”. In this contribution, we integrate molecular analyses, including the plastid sequence data of *I. speculatrix*, together with a re-evaluation of morphological, ecological, karyological, chorological and phylogenetic data on the Hong Kong plant, to describe the new genus *Portiodora*. Three-taxon statement analysis was employed as a primary analytical tool, helping to clarify phylogenetic relationships and support the recognition of *Portiodora*. Two new combinations are established, and an identification key is presented for the “*Iris*-flower clade”. Relationships to other Chinese taxa often related to *P. speculatrix*, such as *I. grijsii*, *I. cavaleriei* and *I. fujianensis* are discussed, and their inclusion in *Portiodora* is not favoured based on the available data. Furthermore, the contribution of taxonomic resolution to biodiversity conservation is discussed.

## 1. Introduction

The genus *Iris* L. (*Iridaceae*), when treated in a broad sense, is one of the most diverse and well-known genera in Asparagales, with nearly 300 species widespread in the Northern Hemisphere [1]. The irises (“rainbow-flowers”) are extremely popular among gardeners and have a high economic impact in the horticultural trade. They are broadly used as ornamental plants throughout the world, but have also been used for medicinal, culinary and industrial purposes since antiquity [2,3]. The group also includes some outstanding model systems in evolutionary biology, particularly those used for studying hybridisation and speciation in plants [4,5,6]. Indeed, forced hybridisation was part of the origin of thousands of garden cultivars of irises [7,8], and processes of reticulate hybridisation and allopolyploidy [9] are also responsible for speciation events in the genus. Morphologically, irises show a broad diversity in both vegetative and floral features, which is often connected to geographical or ecological radiation [6,10,11]. Further, cytogenetic variability is also common in the group, with basic chromosome numbers ranging *x* = 7–12, and with polyploidy, allopolyploidy, disploidy and aneuploidy processes being frequent [12,13].

However, the taxonomy of the irises is quite complex, and the position of some groups remains controversial or not entirely resolved. Molecular work including members of the major taxa (subgenera, sections, etc.) of *Iris* s.l. (the “*Iris*-flower clade” Mavrodiev et al. [14]) produced phylogenies in which morphologically close groups resulted in genetically incoherent classifications [15,16,17]. Additionally, many of the widely accepted infrageneric taxa were revealed to be non-monophyletic; hence, they should not be used as proper taxonomic entities as currently circumscribed [18].

**Figure 1:**
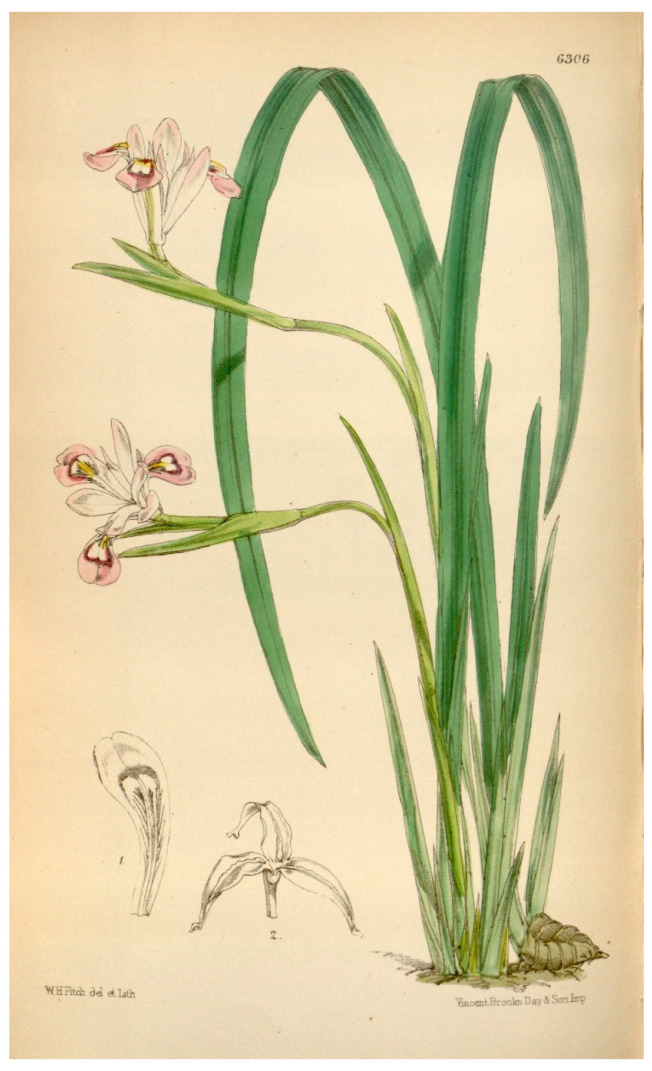
*Iris speculatrix* Hance from the type locality, Hong Kong. Illustration from J.G. Baker [20].

One such contentious topic is the case of *Iris speculatrix*. The species was described by Hance [19] from plants collected “in monte mare prospectante, inter Victoria Peak and Mount Davis” in Hong Kong Island, and was later illustrated by Baker [20] (Figure 1). It is often accepted to extend through a vast area in southeastern China [21], where it is usually referred to as the “small-flower iris” or the “Hong Kong iris”. A more complete description was presented by Zhao et al. [21] in *Flora of China*, which included a drawing (Figure 355, 1–4) available at http://www.efloras.org/object_page.aspx?object_id=60614&flora_id=2 (accessed on 30 September 2025). The species is scheduled under the Forestry Regulations [22] that control the sale and possession of rare plants subject to exploitation, and it is regarded as endangered (EN) [23], according to the categories of the IUCN [24].

A unique morphology allows easy identification of *I. speculatrix*, such as the short rhizome, which is oblique and annulate, tufted or shortly-creeping; the evergreen leaves are linear, long tapering, grassy, somewhat glossy, much overtop the flowers, with several longitudinal ribs (at least the midrib prominent) and cross-veining. The scapes are short, bearing two long pedicellate small flowers that are unscented, ephemeral, and mostly lilac (rarely whitish). The perigone shows a short broad tube at the base. The falls (outer floral segments) and standards (inner floral segments) are rather similar in length and shape; the falls are obovate, often retuse at the apex, with the blade smooth and showing a peculiar dark-violet cordate mark that surrounds a white speckled area connecting with the yellowish to orangish undissected and low ridge-like crest. The standards are somewhat narrower, erect-patent to suberect and clawed. The capsule is patent, with a long beak and three longitudinal ridges, and opens into three valves curving backwards after dehiscence. The seeds are numerous, globose and show a whitish, long appendage resembling a wing on drying (modified from [7,21,25,26]).

Those peculiar features make *I. speculatrix* an enigmatic species that exhibits multiple morphological connections to other congeners. For that reason, some authorities placed *I. speculatrix* in contrasting groups of *Iris* L., such as subg. *Eremiris* Spach [19], or *I.* subg. *Crossiris* Spach (as *I.* subg. *Evansia* (Alef.) Baker) [27], or their respective synonyms at different infrageneric ranks (see [28]). Nonetheless, it is often considered a member of *I.* (subg. *Limniris*) ser. *Chinenses* (Diels) G.H.M.Lawr. [25,26], albeit its morphological and cytogenetic features are said to be deviant.

In the last two decades, molecular work has revealed that *I. speculatrix* constitutes an isolated lineage sister to the beardless/uncrested subgroup of the so-called “*Spuriae-Tenuifoliae* alliance” [29] and also the clade including “subg. *Xiphion*” plus “subg. *Xyridion*” (sensu Wilson [17]), and in any case it is not a member of *I.* (subg. *Limniris*) ser. *Chinenses* (Diels) G.H.M.Lawr. (see [23,28,29,30]).

The comprehensive multi-generic treatment by Crespo et al. [13] did not include molecular data for *I. speculatrix*, and the name was tentatively placed in the genus *Zhaoanthus* M.B.Crespo, Mart.-Azorín & Mavrodiev (*I.* subsect. *Chinenses* Diels) mostly based on morphological affinities and distribution. The treatment recognises up to 25 genera that basically accord with the distinction by horticulturists of the same groups as unranked working names [8]. Those names have traditionally been used during the last two centuries and to the present day, which evidences the readily distinguishable morphology (both vegetative and floral) of each group and supports acceptance at the genus rank.

In that context, the primary aims of the present contribution were: (i) to evaluate the generic position of *Iris speculatrix* in the “*Iris*-flower clade” through an integrative taxonomic approach; and (ii) to discuss the impact of accurately resolving the taxonomy of cryptic biodiversity on its conservation. In order to achieve this, information from new molecular analyses (using both the three-taxon statement and maximum likelihood approaches, hereinafter referred to as 3TA and ML, respectively) that included new plastid sequence data of *I. speculatrix* [23] was integrated alongside a re-evaluation of morphological, ecological, karyological, chorological and phylogenetic data. Accordingly, the generic assignment of the Hong Kong plant is reconsidered and the new genus *Portiodora* is herein described, providing a more complete picture of a multi-generic arrangement of the “*Iris*-flower clade”. Furthermore, the findings of this study suggest that the precise resolution of the taxonomy of cryptic biodiversity may be crucial for effective conservation measures, thus providing a framework for a future protective strategy for the entire “*Iris*-flower clade”.

## 2. Materials and Methods

Morphological data were obtained from observations on both digitised images and vouchers, including type material, from the herbaria A, ANUB, AU, BM, CDBI, CSFI, CSH, CUHK, E, GXMG, HK, IBK, IBSC, IMC, K, KUN, L, LE, NAS, NY, P, PE, QNUN, US, and WELT (acronyms according to Thiers [31]), as well as from the protologues of the concerned names. Over 150 herbarium vouchers were analysed, with a selection of these being presented in Section 3.1.2 as “Other selected material” and including in brackets the herbarium acronym and barcode number assigned to each specimen. Nomenclatural issues conform with the *Madrid Code* (ICN; [32]).

Chromosome numbers presented for the accepted taxa correspond to the first published confident count. Additional data can be accessed in the Chromosome Counts Database-CCDB (https://ccdb.tau.ac.il/Angiosperms/Iridaceae/Iris/; accessed on 3 April 2025).

Sequence data from an accession of *I. speculatrix* collected in Tai Tam, Hong Kong [23] were included in an expanded dataset (206 taxa) based on Mavrodiev et al. [14]. Two vouchers corresponding to the Tai Tam collection labelled “Rare and endangered plants (*S.W. Shek, K.W. Lam, D.T.W. Lau & J.Y.Y. Lau*) 9A & 9B” (respectively HK-0051268 and CUHK-05908) were checked for identity confirmation. Further, additional samples of that species at GenBank (https://www.ncbi.nlm.nih.gov/Taxonomy/Browser/wwwtax.cgi?id=1043429; accessed on 20 July 2024) were added: (i) the accession *Guo CH08–10* from Zhejiang Province [28]; (ii) the accession *ZhouSL-yunnan-Z219* (IBK-00188752) from Guangxi Province [33]; and (iii) the accession *ZhouSL-xingan-Z081* (KUN-310309, barcode KUN-0360549) from Xingan, Jiangxi Province [33]. Vouchers from the latter two, respectively IBK-00188752 and KUN-0310309, were also checked for confirmation. It is worth mentioning that apparently the provenance of the latter two was accidentally permuted in Li et al. [33]. Due to the extremely high sequence identity, we selected the plastome of *I. speculatrix* (accession OK274247) [23] for the current analyses as the most extensive and inclusive option. Accordingly, this accession serves as an analytical placeholder for all currently available plastid sequences of *I. speculatrix*.

Following Mavrodiev et al. [14], we analysed the current cpDNA alignment using the 3TA and ML methods. For corresponding references and details on 3TA, see the legend of Figure S1, Table S1 and Mavrodiev et al. [14]. The ML analysis was conducted in IQ-TREE (http://www.iqtree.org/; accessed on 20 July 2024), as implemented in CIPRES (https://www.phylo.org/; accessed on 20 July 2024). See the legend of Figure S2 for more details. The resulting trees (Figures S1–S3) were interpreted in light of the morphological data of the related clades.

## 3. Results

### 3.1. Taxonomic Treatment

#### 3.1.1. ***Portiodora*** M.B.Crespo, Mart.-Azorín & Mavrodiev ***gen. nov.***

##### Type Species: *Portiodora speculatrix* (Hance) M.B.Crespo, Mart.-Azorín & Mavrodiev

**Diagnosis**: *Genus notabilis Asiae austro-orientalis ab Iride s.str. distinctissimo et praecipue differt rhizomate subsuperficiali, repente, obliquo, brevi, vix incrassato, parce ramoso, tortuoso, annulato, griseo-brunneo, squamoso, reliquiis fibrosis basium foliorum obtecto, quae in fibras crassas rigidas demum mutantibus; foliis sempervirentibus, linearibus, acutis, firmis, nitidis, a basi dense dispositis, margine minute hyalino-serrulato, longitudinaliter nervosis, 1–5 nervis prominulis (praecipue centralibus saepe validioribus) percursis; scapo tereti vel vix compressi, quam foliis multo breviore, interdum arcuato, aliquot submembranaceis bracteiformibus foliis laxe vestito; spathis herbaceis, inaequalibus, lanceolatis, acuminatis, supra carinatis; floribus terminalis, tubo perigonii breve, lato, cylindrico; omnibus segmentis perigonii laevibus imberbibusque, exterioribus majoribus, erecto-patentibus, obovato-spathulatis, lamina subretusa ungue subduplo breviore, apice subfalcata recurva, costa unica centrali laeve subintegra vix prominentula, interioribus erectis vel subpatentibus, parce minoribus, oblancelolatis, obtusis, a basi longe unguiculato-canaliculatis; stylis segmentis exterioribus perigonii subaequantibus vel vix brevioribus, subrecurvatis, anguste triangularibus, ad margines exteriores dentatis; lamella stigmatica integra late subtriangulari, obtusa; capsula patente, oblongo-fusiformibus, ad apicem longe rostrata, subangulosa, transverse striata, e apice deorsum a basi valde dehiscente, valvis divergentibus recurvatisque; seminibus atrofuscis, globoso-angulosis, rugulosis, arillo albido carnoso praeditis, quo in siccitate alam brevem aemulans. Numerus basicus chromosomatum, x = 11. Sola species hujus generis per totam Sinam austro-orientalem invenitur, praesertim in Hongcongum Insula (Portus odorus) et aliis partibus montium propinquorum.*

**Figure 2:**
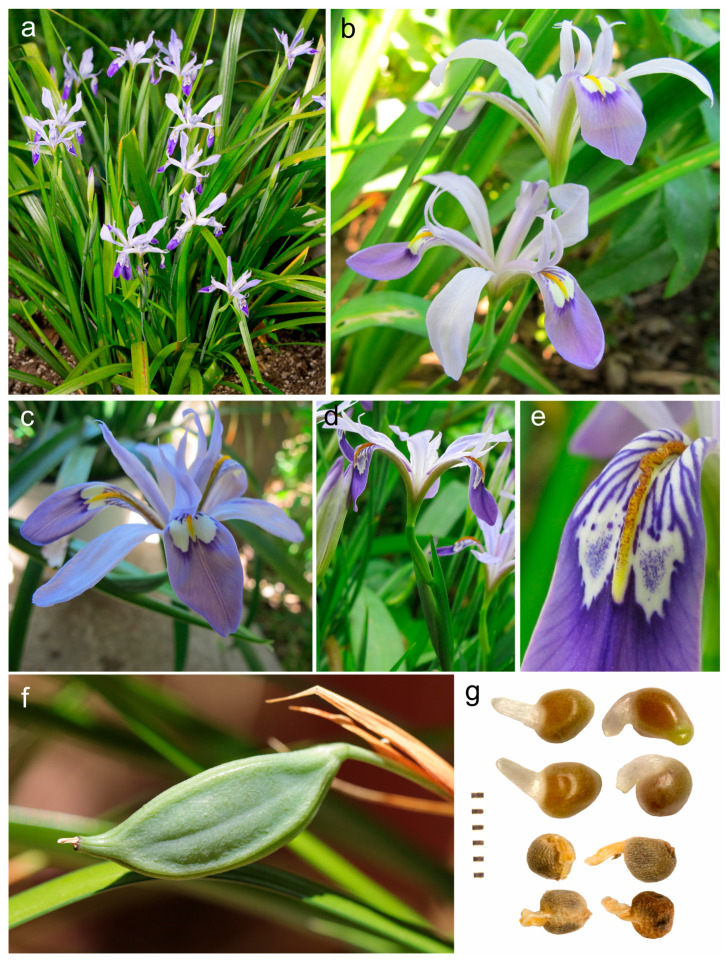
*Portiodora speculatrix*. (**a**) Habit of leaves and flowering stems; (**b**) Detail of flowers; (**c**) Detail of falls, standards and style crests; (**d**) View of ovary and flower tube; (**e**) Detail of crest and ornamentation of falls; (**f**) Beaked capsule; (**g**) Seeds with fleshy appendage (scale in mm). Photos: Kenneth Walker (**a**,**f**–**g**), and Jim Murrain (**b**–**e**).

**Description**: *Rhizomatous herbs*, with evergreen aerial structures. *Rhizome* subsuperficial, oblique, short, few-branched, tufted or short-creeping, slender, tortuous, annulated, greyish brown, coated with fibrous remains of leaf bases that finally turn into rigid fibres; *roots* numerous fibrous. *Leaves* isobilateral, equitant, linear, long tapering, densely disposed at the base, minutely hyaline-serrulate on margins, with 1–5 prominent longitudinal ribs (at least the midrib prominent) and cross-veining, ±glossy on the adaxial surface, much overtopping flowers, not fetid, surrounded by brownish incrassate fibres at base. *Stems* (scapes) aerial, ±arcuate, usually slender and much shorter than the basal leaves, ±terete, bearing several submembranous bract-like leaves. *Flowers* (1–)2, small, terminal on scapes, long pedicellate, unscented, ephemeral, mostly lilac or rarely whitish. *Spathe* valves 2–3, unequal, herbaceous, green, scarcely inflated, ±lanceolate, acuminate, keeled on the upper part. *Perigone* in two rows, ±differing in size and shape, fused in a short broad subcylindric tube; falls erect-patent, larger, broadly obovate-spatulate, patent towards apex, subretuse, gradually tapering into a short claw, smooth, showing a peculiar dark-violet cordate mark that surrounds a white speckled area connecting with the yellowish to orangish undissected and low (ridge-like) crest on the midrib, almost entire and only sinuous in its central section; standards somewhat smaller, suberect to erect-patent, narrowly oblanceolate, acute, narrowed into a long haft. *Stamen* filaments free, adnate to the fall bases. *Ovary* trilocular, with axile placentation. *Style* filiform, with three petaloid branches, almost equalling to slightly shorter than falls, each one concealing a stamen; *crests* long, ±recurved, narrowly triangular, with dentate outer margins; *stigma* broadly subtriangular, obtuse, with entire margins. *Capsule* patent, long exerted above the spathes, oblong-fusiform, acute, subtrigonous, with three weak longitudinal ridges, transversally striate, long-beaked, opening from apex to base with valves curving backwards to expel the seeds upon dehiscing, showing a subcoriaceous pericarp. *Seeds* numerous, globose-angulose, dark brown, with a long, whitish to pale brownish, fleshy appendage resembling a wing on drying; *testa* surface ± rugulose. Basic chromosome number: *x* = 11. Figure 1 and Figure 2.

**Etymology**: *Portiodora* derivates from “Portus odorus”, which is the Latin adaptation for “the fragrant harbour”, a name formerly applied to Hong Kong that is literally the phonetic translation of its Cantonese name (“香港”). It thus honours the type locality of *P. speculatrix*, the first species described within this enigmatic lineage.

#### 3.1.2. ***Portiodora speculatrix*** (Hance) M.B.Crespo, Mart.-Azorín & Mavrodiev ***comb. nov.*** [≡ *Iris speculatrix* Hance in J. Bot. 13: 196 (1875), Basionym]

**Figure 3:**
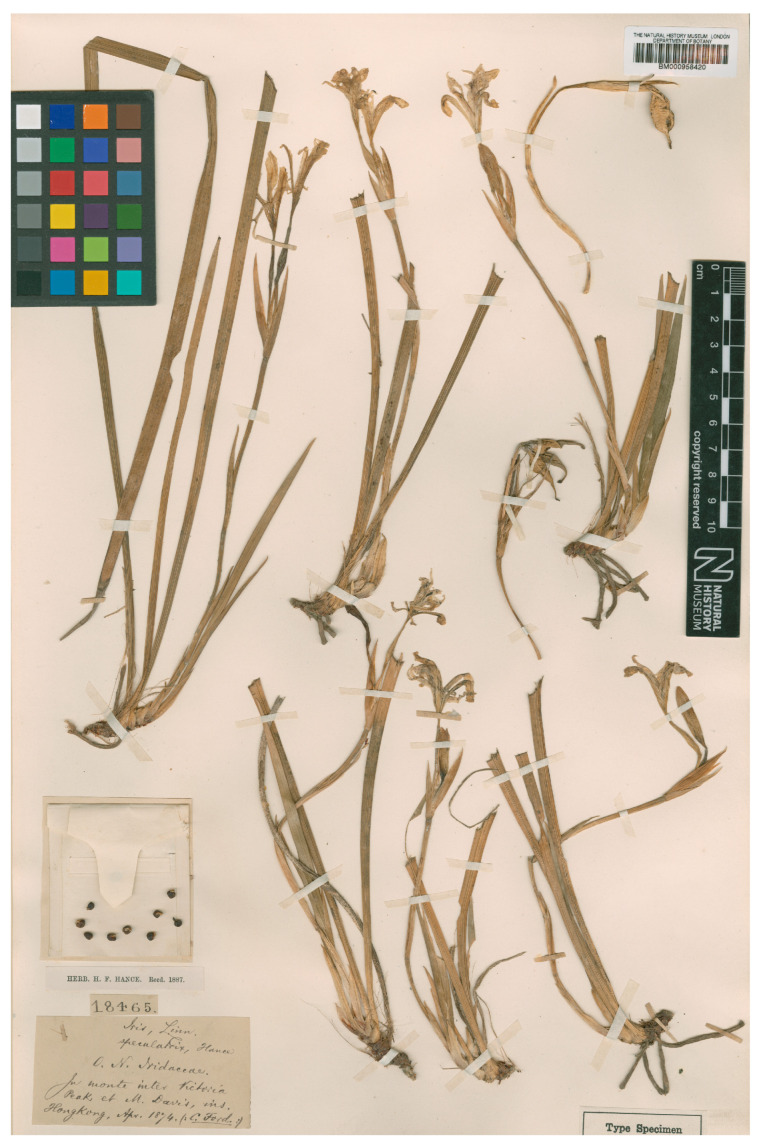
Lectotype (designated here) of *Portiodora speculatrix* (*Iris speculatrix*) housed in the British Museum Natural History, London (BM-000958420).

**Lectotype (designated here)**: “In monte inter Victoria Peak et M. Davis Apr. 1874, C. Ford”, ex Herb. H.F. Hance n.º 18465 (BM-000958420 https://data.nhm.ac.uk/media/60d9e994-be9b-47a1-a64b-d0e570cdaf4b, (accessed on 30 July 2025)), Figure 3. Isolectotypes: K-000499078 [digital image]: https://images.data.kew.org/image/d0750f29-61f4-4fb0-9540-c0562c34185d, (accessed on 30 July 2025); PE-00034060 [digital image]: https://www.cvh.ac.cn/spms/detail.php?id=04805f06, (accessed on 30 July 2025); PE-01013509 [digital image]: https://www.cvh.ac.cn/spms/detail.php?id=ef698537, (accessed on 30 July 2025).

**Other selected material**: CHINA. **Hong Kong SAR**: Hong Kong, indigenous on peak to eastward of Mt. Davis, April 1874, *C. Ford* (K-002930862); Hong Kong, ravine Mount Victoria, April 1874, *Mr. Ford* (L-1473474); Hong Kong, hill above Repulse Bay [Tsin Shui Wan], 13 April 1900, *W.J. Tutcher 669* (K-002930857); Hong Kong, 29 April 1930, *Z. Jinglie 21659* (IBSC-0628992, IBSC-0628994); Hong Kong, Cape d’Aguilar, 3 May 1970, *S.Y. Hu 10051* (A-00962200, K-002930860, PE-01013510, US-2732045); Hong Kong, Violet Hill, 21 April 1992, *S.Y. Hu & P. But 20422* (A-00962199, L-3708985); Hong Kong Island, Dragon’s Back, May 1970, *D.P.M. Guile 2519* (K-002930854); idem, *D.P.M. Guile 2520* (K-002930855); Hong Kong, Tai Tam, 172 m elev., 20 April 2021, *S.W. Shek* et al. (*R9A*: HK-0051268; *R9B*: CUHK-05908, HK-0051269). Lantau Island, 9 May 1888, *Native Coll. 53/88* (K-002930861) **Anhui Province**: Chishi, Shitai, Shuanghekou Nature Reserve, sampling track number 3, at the starting point, 273 m elev., 11 August 2012, *H. Xin-Zhou SB0206* (KUN-1393279); Chizhou, Shitini County, Baniajiang Nature reserve, 221 m elev., 11 May 2017, *S.J.L. Jin ANUB01248* (ANUB-001536); Jiuhua, Wubian, Shouyang, 200 m elev., 7 May 2008, *L. Pan LP0805061* (CSH-0073241). **Chongqing Province**: Nanchuan mountain, spring, 800 m elev., 20 May 1980, *L. Wei 300* (CDBI-0169631). Nanchuan District, Jinfo Shan Nature Reserve, 720 m elev., 20 May 1996, *Z. Liu 17140* (PE-02238585); Jinfo Shan Mountain, 1500 m elev., 12 July 1990, *L. Zengyu 901428* (IMC-0013888). **Fujian Province**: Kuliang hills, near Foochow [Fuzhou], 300–900 m elev., July–August 1919, *J.B. Norton 1230* (US-1050954); Inghok Hsien, Tahshian, in the shade of bamboo, 20 April 1924, *H.H. Chung 2618* (K-002930859); Wuyi Mountain, 9 May 1983, *Matsumoto 8305054* (AU-032605); Da’anyuan, Wuyishan, 516 m elev., 30 April 2016, *H. Xueliang* et al. *2016043029* (AU-054672). **Guangdong (Canton) Province**: Kwangtung, Lokchong, 18 May 1929, *C.S. Tso 20615* (PE-01013507, NY-04354496, NY-4354499); Kwangtung, Loh Fau Shan (Loh-Fau Mountain), 7 May 1917, *C.O. Levine 701* (PE-01604321); Huahu village, 500–600 m elev., 12 May 1957, *D. Liang 4764* (IBSC-0628954, NAS-00555473, PE-01013504). **Guangxi Province**: Guilin, Guanyang, Dongbing, Baoliang village, Forest of the wild boars, 69 m elev., 9 May 2016, *Guanyang Censing Team 450327160509007LY* (GXMG-0173704); Rong’an County, Tantou, Hedong, 211 m elev., 11 April 2019, *Rong’an Censing Team 450224190411024LY* (IBK-00421700); Zhuang region, 31 May 1979, *L. Guangguang 63313* (IBK-00188751, IBK-00188752). **Guizhou Province**: Daozhen, 830 m elev., 20 May 1996, *L. Zhengyu 20896* (PE-02238558); Guizhou, 420 m elev., 24 April 2018, *L. Jiaojiao 20183547* (QNUN-0025878); Zhizhen, 750 m elev., 25 April 1996, *L. Zhengyu 20897* (PE-02238560). **Hunan Province**: Xining County, 500 m elev., 5 July 1995, *L.B. Luo 0867* (E-00071325, US-3524938, WELT-074625); Pingxi village, Huangsang Nature Reserve, 22 May 2013, *Z.J. Zhou Dian 13388* (CSFI-028930, CSFI-028931, CSFI-028932); Dong’an county, 180 m elev., 6 May 2014, *Y.X. Liming 14050601* (CSFI-028934). **Jiangxi Province**: Wuning County, Pingyang Mountain, Bamboo Forest Farm, 350–450 m elev., 7 August 1995, *Y. Cun-su 333* (NAS-00600599); Ruijin County, Xiqi, 15 July 1958 *H. Qiming 3572* (IBK-00251328, PE-00034064). **Zhejiang Province**: Tianmu Shan (Tianmu Mount), near the Zen Temple, 380 m elev., 9 June 1957, *D.M. Bin 4314* (PE-01013456); Linan Shi, Tianmushan, Laodian, 1082 m elev., 29 April 2010, *J. Wen* et al. *11312* (US-3625658); Lin’an County, Xitianmu Mountain, 9 July 2014, *C. Bin* et al. *WZW01097* (CSH-0009888); Quzhou Jiangshan, Hongyanding, 11 May 2018, *Y. Yuehong* et al. *CFH09001954* (CSH-0156163); Tihtaishan (Tihtai Mountain), 600–1200 m elev., 5–18 May 1924, *R.C. Ching 1446* (P-02162379, US-1246353). Uncertain location: **Yunnan Province**: Yunnan, *Liu* (PE-01013519, PE-01013520, PE-01013521) [Although the specimens appear to match *P. speculatrix*, all three vouchers lack precise collection details, which makes site location difficult. New and reliable collections are required to verify the presence of the species in this province].

**Description**: For a detailed description of the species, which complements the generic description presented above, see Waddick and Zhao [26] and Zhao et al. [21].

**Chromosome number and formula**: 2*n* = (4*x*) = 44 (4M + 24m + 14sm + 2sm^sat^), with all chromosomes quite homogeneous and similar in size [34,35,36,37].

**Figure 4:**
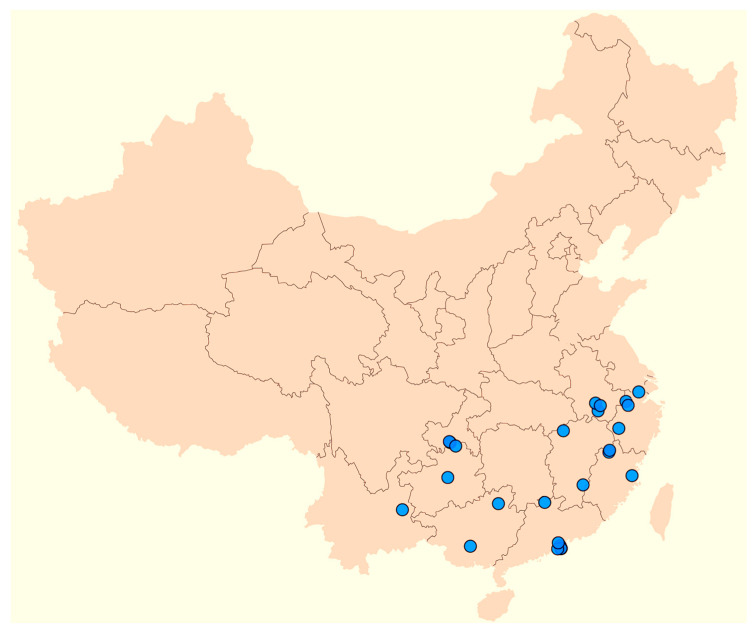
Distribution of *Portiodora speculatrix* in China (circles), based on the studied material.

**Ecology and distribution**: *Portiodora speculatrix* usually occurs in organic matter-rich substrates of forest fringes, open groves and moist grasslands. It is also sometimes found in open windy and rocky sites, often facing the sea. It ranges from sea level up to about 1200–1500 m elevation. Described from Hong Kong Island, it extends into the neighbouring areas of southeastern China (Figure 4), namely the provinces of Anhui, Chongqing, Fujian (Fokien), Guangdong (Canton), Guangxi, Guizhou, Hunan, Jiangxi and Zhejiang, according to our data (see also [21,25,38,39,40,41] and the Selected Studied Material, above). Flowering: April–May; fruiting: July–August.

**Infraspecific variation**: Taxonomically, two flower-colour variants have been recognised [21]: *Iris speculatrix* var. *speculatrix* for the typical lavender- to light violet-flowered individuals (the typical form), and *I. speculatrix* var. *alba* V.H.C.Jarrett for the white-flowered (albino) individuals [42]. However, plants of both types coexist in a single population [42], and therefore they are regarded here as mere forms. Consequently, a new combination is established below to accommodate both names in the new genus:

***Portiodora speculatrix*** f. ***speculatrix***.

***Portiodora speculatrix*** f. ***alba*** (V.H.C.Jarrett) M.B.Crespo, Mart.-Azorín & Mavrodiev ***comb. nov.*** [≡ *Iris speculatrix* var. *alba* V.H.C.Jarrett in Sunyatsenia 3: 265 (1937), basionym]. Type: “Hong Kong: Violet Hill, eastern slopes, 19 April 1937, growing amid the blue type forms; type in the Hongkong Botanic Garden” [42].

**Figure 5:**
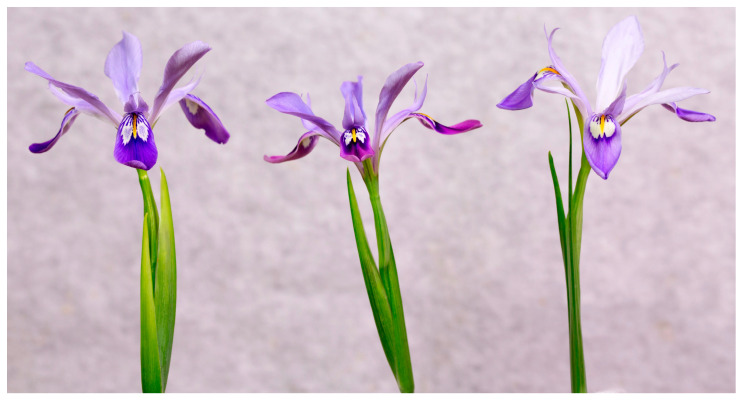
Flower colour patterns in three cultivated clones of *Portiodora speculatrix* (left: “John Lonsdale clone” of uncertain origin; centre: “Darrel Probst clone” from Sanmen, Zhejiang Province; right: “Shanghai Waddick clone” from Shanghai Botanical Garden). Photo: Kenneth Walker.

At present, not less than three clones of *P. speculatrix* are cultivated by horticulturists [43]: the “John Lonsdale clone” of uncertain origin, the “Darrel Probst clone” from Sanmen (Zhejiang Province), and the “Shanghai Waddick clone” (Figure 5). The specimens are consistent with the protologue and current concept of the species, and they only appear to differ in some features concerning the ornamentation and colour of the falls and the size of the spathe. Therefore, those clones should be considered to be part of the intraspecific phenotypic variation of the type form of the species. For data on floral syndrome and breeding systems of the species see Li et al. [38].

**Conservation**: The majority of the studied collections are dated before 1990, and *P. speculatrix* might be in decline or extinct in many sites. In the known localities, the species is subject to significant anthropogenic pressure and is primarily confined to natural reserves and protected areas. On this basis, the species has been evaluated as EN [23] in accordance with the IUCN [24] categories. New comprehensive data are needed to clarify this point.

#### 3.1.3. Comparative Molecular and Phylogenetic Results

**Figure 6:**
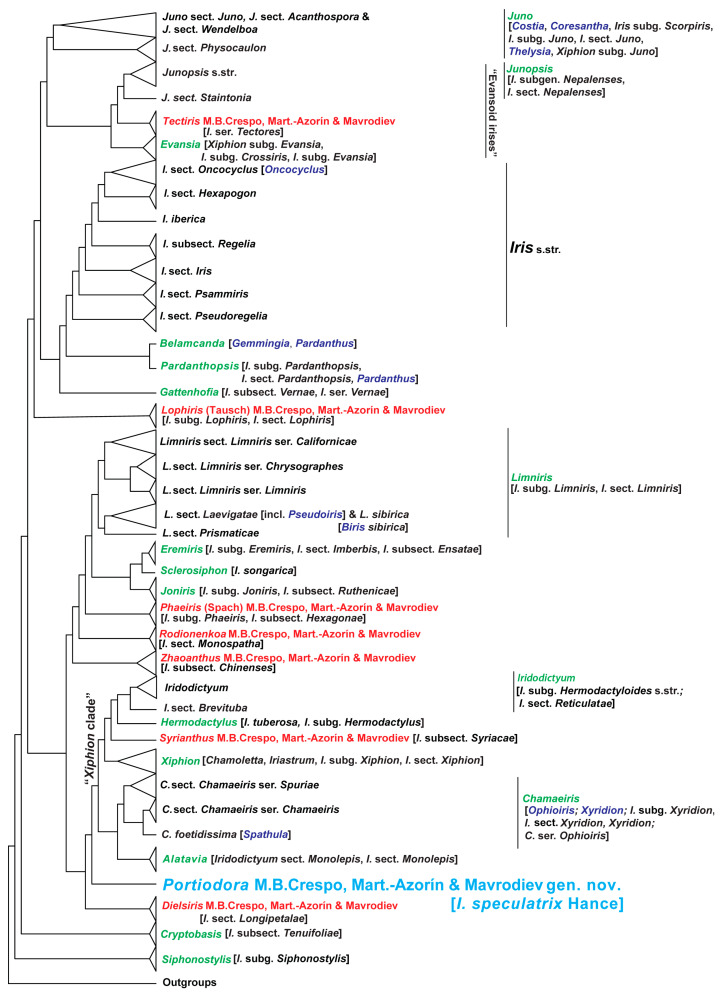
Summarised relationships within the “*Iris*-flower clade”: evidence from three-taxon statement analysis. Cladogram representing the summary of hierarchy of patterns resulted from 3TA of cpDNA sequence data (Figure S1). In addition to the information presented in Figure S1, the colour-coding scheme summarises data on genera that have been restored (green font), recently described (red font), or rejected (purple font) by the authors of the present study over the past decade of research. The name of the new genus *Portiodora*, described in this article, is shown in light blue font. The synonymy provided in square brackets is necessary but not exhaustive. Figure S3a partially represents a simplified version of Figure 6.

**Figure 7:**
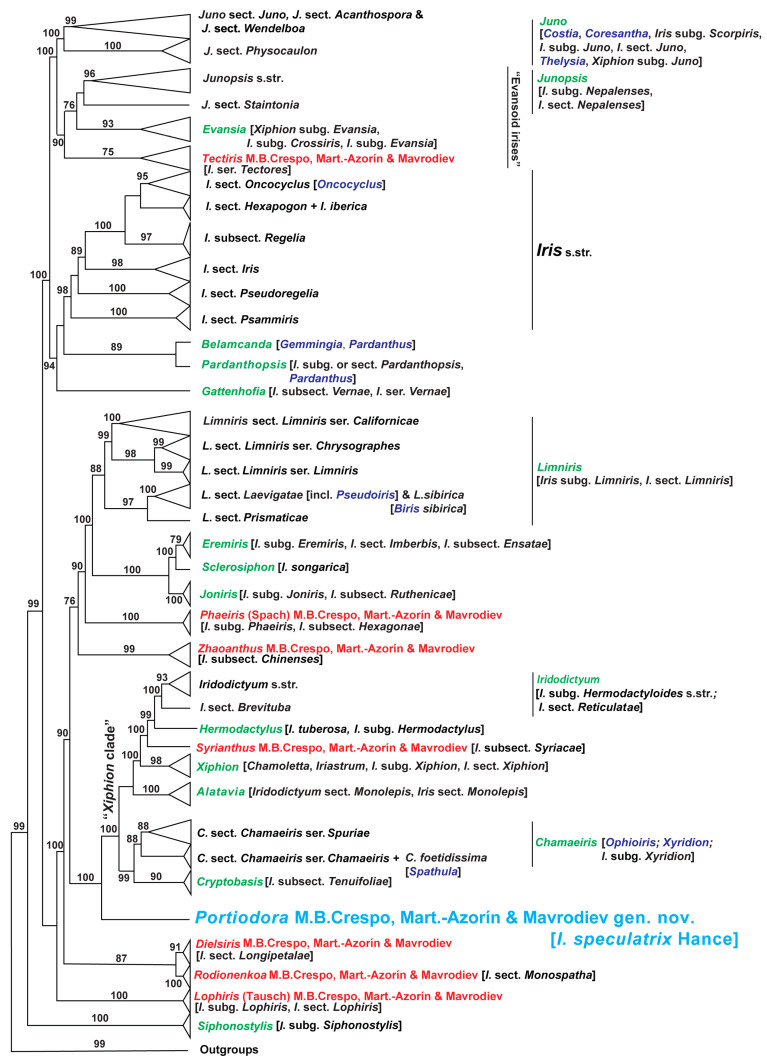
Summarised relationships within the “*Iris*-flower clade”: evidence from Maximum Likelihood analysis. Tree representing the summary of conventional molecular ML analysis of the cpDNA sequence data (Figure S2). The approximate Likelihood Ratio Test (aLRT) values (see Mavrodiev et al. [14] for references) were rounded to the nearest whole number and shown above or below branches when equal to 75% or higher. All clades corresponding to accepted genera, as well as most deep branches received high statistical support as assessed by the aLRT. See the legend of Figure 6 for the description of the colour-coding scheme. The synonymy provided in square brackets is necessary but not exhaustive. Figure S3b represents a partially simplified version of Figure 7.

The 3TA cladogram (Figure 6) summarises the hierarchy of patterns (Figure S1) with a score of 23, representing the Robinson–Foulds (RF) median consensus of eight cladograms. Each cladogram corresponds either to a single most parsimonious tree or to the RF median consensus derived from the set of most parsimonious trees obtained through 3TA (see Table S1 and Mavrodiev et al. [14] for details and references). Only informative conventional DNA characters were included in the 3TA analyses, and the values for the operational outgroup were fixed based on the 50% majority-rule consensus of the initial conventional DNA alignment (supermatrix; 202 terminals, see Table S1 for the number of the three-taxon statements in each of eight analyses) or a subset of it. Unlike ML analysis, statistical support in 3TA is optional and not required for validating the hierarchy of patterns, as this method is not focused on probabilistic measures of confidence (see Mavrodiev et al. [14] for references). The ML cladogram (Figure 7) represents the most probable and well-resolved maximum likelihood tree (−ln likelihood = 48,909.1674) (Figure S1), inferred using IQ-TREE from a plastid supermatrix of conventional *Iris* s.l. and outgroup taxa (206 terminals, 8646 nucleotide sites). The best-fit model, TVM + F + I + G4, was automatically selected by IQ-TREE according to the Schwarz (Bayesian) Information Criterion. Statistical support was assessed using the approximate Likelihood Ratio Test (aLRT) (see Mavrodiev et al. [14] for references).

The general topology and internal relationships of clades in both trees match the results previously obtained by Mavrodiev et al. [14] from a narrower database (173 ingroup plus five outgroup taxa). The 3TA placed *Portiodora speculatrix* as a sister to the *Xiphion* s.l. clade (Figure 6 and Figure S1). At the same time, it shows that *Zhaoanthus* is nested in a completely distinct level of the obtained hierarchy of patterns, as a sister of the broad clade that includes the subclades corresponding to the genera *Rodionenkoa* M.B.Crespo, Mart.-Azorín & Mavrodiev, *Phaeiris* (Spach) M.B.Crespo, Mart.-Azorín & Mavrodiev, *Joniris* (Spach) Klatt, *Sclerosiphon* Nevsky, *Eremiris* (Spach) Rodion., and *Limniris* (Tausch) Rchb. (Figure 6 and Figure S1). The ML analyses also recovered *P. speculatrix* as a strongly supported sister to the *Xiphion* s.l. clade (100% aLTR) (Figure 7 and Figure S2). The monophyly of the *Xiphion* s.l. clade itself (after *P. speculatrix*) also received strong statistical support (100% aLTR) (Figure 7 and Figure S2). Furthermore, *Zhaoanthus* forms a well-supported clade (100% aLTR) thus only distantly related to *Portiodora*. The general placement of *Zhaoanthus* within the ML tree is similar to that obtained in 3TA, except for the distant relationship with *Rodionenkoa* (Figure 7 and Figure S2). This placement of *P. speculatrix* as a sister to the *Xiphion* s.l. clade (Figure 6, Figure 7, Figures S1 and S2) is strongly incongruent with morphology-based classifications that placed the species in *Zhaoanthus*. The internal relationships in this clade are congruent in both analyses, but not entirely identical. The 3TA cladogram (Figure 6 and Figure S1) recovers a *Xiphion* s.l. clade composed of a first subclade including *Iridodictyum* Rodion., *Hermodactylus* Mill., *Syrianthus* M.B.Crespo, Mart.-Azorín & Mavrodiev, and *Xiphion* Mill. sister to a second subclade with sisters *Chamaeiris* Medik. and *Alatavia* Rodion. (Figure 6 and Figure S1). At the same time, 3TA placed *Cryptobasis* Nevsky as a sister of most major clades of *Iris* s.l., close alongside *Siphonostylis* Wern.Schulze (Figure 6 and Figure S1). The ML tree (Figure 7 and Figure S2) includes a *Xiphion* s.l. clade with a first subclade (100% aLTR) comprising *Iridodictyum*, *Hermodactylus and Syrianthus*, which is a successively well-supported (98–100% aLTR) sister to a second subclade with *Xiphion* s.str. and a third one with *Alatavia* (100% aLTR); all of those subclades are positioned as non-supported sisters to a fourth one (*Chamaeiris* and *Cryptobasis*) (99% aLTR) (Figure 7 and Figure S2).

## 4. Discussion

### 4.1. Taxonomic and Phylogenetic Relationships of Portiodora

The phylogenetic placement of *Portiodora speculatrix* (*I. speculatrix*) in the obtained trees (Figure 6, Figure 7, Figures S1 and S2) is fully congruent with the results of previous molecular systematic studies focused on two [15] or five [28] plastid markers (respectively, *rps4* plus *trnL-trnF*, and *matK/trnK*, *trnL-trnF*, *psbJ–petA*, *rpl32–trnL*, plus *rpoB–trnC*), and based on a more reduced but rather comprehensive sectional dataset. These authors, however, found that *I. speculatrix* was highly sequence-divergent from the remaining members of the beardless/uncrested subgroup of the so-called “*Spuriae-Tenuifoliae* alliance” (or also the “*Xiphion* s.l. clade”) and concluded that their phylogenetic position was congruent with their historically problematic taxonomic adscription in *Iris* (s.l.). Identical results were recently reported by Siu et al. [23] and Choi and Lee [30] based on whole plastome phylogenies, the latter authors presenting a rather complete tree covering most of the major groups of *Iris* (s.l.). All these findings thus corroborate the placement of *Portiodora* far from members of *Zhaoanthus* (*I*. subsect. *Chinenses*) and other crested irises [23,28,29,30] to which it has sometimes been connected [13,25,26]. Further, beside the molecular evidence some morphological, anatomical and chromosomal features well support the current basal placement of *I. speculatrix* regarding the *Xiphion* s.l. clade (see [13,29,44]).

### 4.2. Morphological Connections of Portiodora to Other Major Groups of Irises

In the protologue of *Iris speculatrix*, Hance [19] related his new species to *I. oxypetala* Bunge and *I. biglumis* Vahl (both often included in *I. lactea* Pall.), which are regarded here to belong to *Eremiris*. Although some morphological similarities exist, *Eremiris* and *Portiodora* differ significantly in terms of their floral structures (e.g., the outer petals of *Eremiris* are acute, lacking a central yellow crest but provided with a low entire keel), stigma features (*Eremiris* has long and acute stigmatic lips), and fruit and seed morphology (*Eremiris* produces erect, obtuse capsules with six weak nerves and a short week, and its seeds lack fleshy appendages). These divergences support their phylogenetic placement far from each other (Figure 6 and Figure 7).

*Portiodora* evidently differs from *Eremiris* but also from most members of the “*Iris*-flower clade”, and it is easily distinguishable by a unique morphology (Table 1). At first glance, *P. speculatrix* approaches to some extent species of *Zhaoanthus* (*I.* subsect. *Chinenses*), with which it shares several characteristics, such as the evergreen glossy leaf habit (Figure 2a), the subtriangular entire stigmatic lip, and the arillate seeds becoming winged (appendage ca 1–2 mm long) on drying (Figure 2g). These shared characteristics led Crespo et al. [13] to tentatively include the Hong Kong iris in the latter genus.

However, *Portiodora* considerably differs from *Zhaoanthus* and other Chinenses irises by the more robust rhizome ca. 10–15 mm in diameter, sparingly branched (vs. filiform, up to 5 mm in diameter and often stoloniferous), bearing stronger roots; the outer segments of the perigon (P) subequal or slightly exceeding the style branches (S) in a ratio P/S = 1.0–1.3 (Figure 2b–d) (vs. twice longer, in a ratio P/S = 1.9–2.1); the falls with a single low central smooth and subentire (ridge-like) crest (Figure 2e), lacking lateral outgrowths (vs. crest prominent and markedly wavy, toothed or fringed, flanked with lateral outgrowths); and the patent (ca 70–90° regarding the stem axis) and long-beaked (beak ca 8–14 mm long), fusiform capsules (Figure 2f), which curl the valves backwards and thus expel the seeds upon dehiscence. These fruit features are distinctive in the entire “*Iris*-flower clade”, and the dehiscence type is highly unusual. Only species of *Joniris*, *Chamaeiris foetidissima* (L.) Medik. and *Belamcanda chinensis* (L.) Medik. exhibit similar dehiscence mechanisms, albeit noteworthily the two latter produce coloured, somewhat fleshy, berry-like seeds that are said to be related to ornithochory [13].

All those features connect *P. speculatrix* to some beardless/uncrested species (the *Xiphion* s.l. clade, see above) such as *Chamaeiris* (the “Spuriae”), *Xiphion* Mill. (the “Xiphiums”), *Syrianthus* (the “Syriacae”), *Hermodactylus*, *Alatavia*, *Iridodictyum* (the “Reticulatas”; see [44]), and *Cryptobasis* (the “Tenuifoliae”) (Figure 6 and Figure 7). However, including *P. speculatrix* in any of the available genera seems unrealistic and forced, the same as placing it in any of the extant subdivisions of *Iris* s.l.

Beside the coloured patches or marks on the adaxial face of the falls, the presence of distinct three-dimensional structures such as trichomes, ridges and/or crests is a sort of embellishment that plays an important role in the reproductive biology of irises by directing the pollinator to walk into the pollination tunnel [45,46]. In a study on the “crested irises” usually included in *I.* subg. *Crossiris* by iridologists, Guo and Wilson [28] found that at least the presence of a crest on the fall blade is a homoplastic character that evolved at least five times in the “*Iris*-flower clade”. Among them, the crest morphology of *Portiodora speculatrix* is notably divergent from those in other cristate irises [47,48], such as members of the so-called “core-crested” clade that is formed by four lineages, i.e., *Evansia* Salisb. (*I.* subg. *Crossiris*), *Tectiris* M.B.Crespo, Mart.-Azorín & Mavrodiev (*I.* ser. *Tectores* Rodion.), *Junopsis* Wern.Schulze (*I.* sect. *Nepalenses* Lynch) and *Juno* Tratt. (*I.* subg. *Scorpiris* Spach). But it is also far from the remaining three other crested clades, i.e., *Lophiris* (Tausch) M.B.Crespo, Mart.-Azorín & Mavrodiev (*I.* sect. *Lophiris* Tausch), *Rodionenkoa* (*I.* sect. *Monospatha* Rodion.) and *Zhaoanthus* (*I.* subsect. *Chinenses*), which indicates that *P. speculatrix* originated independently from those other lineages of crested irises [28]. Indeed, the crest of *P. speculatrix* was described as Type I and appears to be lower, less showy and less sophisticated than most of the remaining crested irises [48]. This type is characterised by being only a little higher and more elaborately shaped at its medial segment [47], somewhat approaching the crests in *Zhaoanthus* but lacking the lateral rows of protuberances usually present in the latter. The absence of lateral outgrowths flanking the central crest of *P. speculatrix*, together with a weak development of the distal part of the crest, morphologically recalls the single central entire crest of *Rodionenkoa* or the low entire keel (not a crest) of *Joniris*, *Eremiris* and *Phaeiris* (all three from the “*Limniris* I clade” sensu Wilson [17]) and some members of the *Xiphion* s.l. clade (the subg. *Xiphium* + subg. *Xyridion* clade sensu Wilson [17]). The position of *Portiodora speculatrix* as a basal lineage of the *Xiphion* s.l. clade in the published phylogenies of the “*Iris*-flower clade” appears to be congruent with the crest peculiarities of the species.

For a more detailed morphological comparison of *Portiodora* against the rhizomatous genera in the “*Iris*-flower clade” to which it has often been related, refer to Table 1. Further data are also available from Crespo et al. [13] and Appendix A.

### 4.3. Karyological Remarks

The diploid count 2*n* = 44 has been repeatedly reported for *P. speculatrix* [34,35,36,37], and it can be considered as a tetraploid (4*x*) level in *Iris* (s.l.) from the basic chromosomenumber *x* = 11. This number is found in several lineages of the “*Iris*-flower clade” [13], such as *Iris* sect. *Iris* (i.e., *I. aphylla* L. p.p., *I. billotii* Foster, *Iris florentina* L., *I. germanica* L., *I. kashmiriana* Dykes, *I. kochii* Kerner ex Stapf, etc.), *I.* sect. *Regelia* (Foster ex Baker) Lynch (i.e., *I. hoogiana* Dykes, *I. korolkowii* Regel, *I. stolonifera* Maxim., etc.), *Chamaeiris* (i.e., *Ch. carthaliniae* (Fomin) M.B.Crespo, *Ch. halophila* (Pall.) M.B.Crespo, *Ch. lilacina* (Borbás) M.B.Crespo, *Ch. sogdiana* (Bunge) M.B.Crespo, *I. violacea* (Klatt) M.B.Crespo, etc.), *Phaeiris* (i.e., *Ph. brevicaulis* (Raf.) M.B.Crespo & al. p.p, *Ph. giganticoerulea* (Small) M.B.Crespo & al., *Ph. hexagona* (Walter) M.B.Crespo & al., *Ph. savannarum* (Small) M.B.Crespo & al., etc.), or *Zhaoanthus* (i.e., *Z. proanthus* (Diels) M.B.Crespo & al. s.l.). However, the karyogram of *Portiodora speculatrix* reported by Chimphamba [36] for samples from Hong Kong shows unique peculiarities among the other relatives with similar chromosome number, and it also differs considerably from the karyogram of other cristate irises (Table 2).

Indeed, *P. speculatrix* shows a formula 2*n* = 44 = 4M + 24m + 14sm + 2sm^sat^, with all chromosomes quite homogeneous and similar in size. The karyogram is perhaps closer to that of *Lophiris lacustris* (Nutt.) M.B.Crespo & al. (≡ *I. lacustris* Nutt.), a plant with 2*n* = 42 chromosomes, of which one pair are also satellited but the other four pairs are notably smaller than the rest. This North American species is however quite distant in morphology, biogeography and phylogenetic position from *P. speculatrix.* The connections are weaker to other groups with which it was related, namely the crested irises. In fact, according to data presented in Chimphamba [36], chromosomes with secondary constrictions are typically present in *Evansia* and telocentric chromosomes are found in both *Evansia* and *Rodionenkoa.* All of those types are lacking in *P. speculatrix* and their karyograms differ much in structure. Similarly, Choi et al. [9] and Kim et al. [49] showed that other crested groups such as *Zhaoanthus* show non-satellited chromosomes that are markedly heterogeneous in size, with several pairs of subtelocentric chromosomes in some species such as *Z. koreanus* (Nakai) M.B.Crespo & al., and quite distinct from *Portiodora*. Additionally, other non-crested groups such as *Joniris* (the “Ruthenicae irises”) and *Eremiris* (the “Ensatae irises”), to which *Portiodora* was sometimes related, show chromosomes subhomogeneous in size but smaller, of which one pair is satellited. First, the karyogram of *Joniris* includes tiny chromosomes, with two pairs slightly larger than the rest. In contrast, *Eremiris* shows two pairs of subtelocentric chromosomes that are slightly smaller than the rest [9,49]. Connections to other lineages with 2*n* = 44 in both the *Iris* s.str. clade and the *Xiphion* s.l. clade are perhaps weaker. Species of *Iris* s.str. such as *I. florentina* L. (*I. albicans* Lange) show a heterogeneous karyogram with several pairs of subtelocentric and/or telocentric chromosomes, sometimes with a satellited pair [50]. Conversely, species of *Chamaeiris* such as *Ch. violacea* (*I. musulmanica* Fomin) exhibit a heterogeneous karyogram with only metacentric (26 pairs) and submetacentric (18 pairs) chromosomes, lacking satellites (see [51] as *I. spuria* s.l.). Regarding *Phaeiris* (the “Louisiana irises”), its members often show a heterogeneous karyogram characterised by one long pair (metacentric or submetacentric) and one short pair (subtelocentric) of marker chromosomes, often with secondary constrictions [52]. In some cases (i.e., *Ph. giganticaerulea*), the long pair is absent and the karyogram looks more homogeneous in chromosome size. Those marker chromosomes are accompanied by several pairs of submetacentric and subtelocentric pairs, of which 2–3 pairs of any type are satellited [52]. This conforms overall a quite different karyogram regarding *Portiodora*. All those karyological differences make the newly proposed genus a remarkable distinct lineage regarding other groups with 2*n* = 44 chromosomes.

### 4.4. Ecological and Biogeographical Aspects

*Portiodora speculatrix* is one of the few iris species outside the Chinenses group which thrives in the mesic environments, sometimes open areas, of tropical forests [26]. It is generally accepted that the species is widespread in most of central, southern and eastern China, occurring in a broad range of elevations between 500 and 1800 m [21]. However, the herbarium material we studied was collected in the lower or medium elevation lands of the southeastern provinces (i.e., Anhui, Chongqing, Fujian, Guangdong (Canton), Guangxi, Guizhou, Hunan, Jiangxi and Zhejiang) of the country, from sea level to about 1200–1500 m elevation (Figure 4). References to other Chinese inland and high mountain areas (see [21,26]) correspond to different species such as *Eremiris lactea* (Pall.) Rodion. (≡ *Iris lactea*), *Junopsis decora* (Wall.) Wern.Schulze (≡ *Iris decora* Wall.) and related taxa, or also to species of *Zhaoanthus*, perhaps *Z. probstii* (C.A.Wilson) M.B.Crespo, Mart.-Azorín & Mavrodiev (in press), according to Murrain [41] and Wilson [40]. Although the species was cited in Taiwan [41], no material from that area was studied. Citations from other territories are in need of further confirmation.

### 4.5. The Case of Iris grijsii., I. caveleriei and I. fujianensis

**Figure 8:**
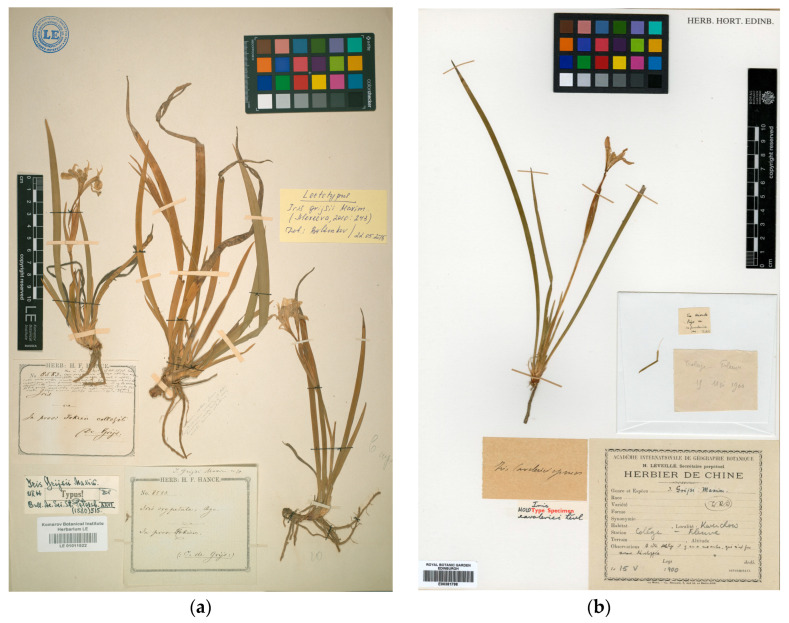
Type specimens of: (**a**) *Iris grijsii* Maxim. (lectotype: LE-01011522); and (**b**) *I. cavaleriei* H.Lév. (holotype: E-00381798).

*Portiodora speculatrix* has frequently been regarded [7,21,25,26] as the earlier synonym of both *Iris grijsii* Maxim. (*de Grijs 8583*; lectotype: LE-01011522, isolectotype: P-01840540; see [53]) and *I. cavaleriei* H.Lév. (holotype: E-00381798) (Figure 8). The latter two species are also native to China, where they entirely overlap in distribution and share morphological features with *Portiodora*. In this respect, Wilson [40] discussed relationships among all three species when describing both *I. probstii* C.A.Wilson and *I. dabashanensis* C.A.Wilson. According to this author and the protologues of *Iris grijsii* [54] and *I. cavaleriei* [55], both species show some shared characteristics, such as the presence of reduced short leaves at the scape base (apparently often absent in *P. speculatrix*, but see PE-01013510; the reliability of this character is to be checked in wild populations), larger floral bracts up to 12 cm long (vs. 2.5–8 cm in *P. speculatrix*), and broader flowers with a perigone tube longer than 10–15 mm (vs. 5–8 mm in *P. speculatrix*). For the aforementioned reasons, Wilson [40] concluded that *P. speculatrix* is not conspecific with either *Iris grijsii* or *I. cavaleriei*, despite the close relationship between these. She also outlined the features distinguishing both of the latter species, such as the leaf width (0.4–1 cm in *I. grijsii* but less than 0.4 cm in *I. cavaleriei*), lower bract length (4.5–7.5 cm in *I. grijsii* but 9–12 cm in *I. cavaleriei*) and flower exertion (flower not exerted above the bracts, albeit visible in *I. grijsii* but exerted in *I. cavaleriei*). In our opinion, and according to Wilson [40], all these characters point to an independent taxonomic treatment of each name.

Furthermore, Maximowicz [54] described the falls of *I. grijsii* (Figure 8a) as being provided with a lower crest (or perhaps, a raised ridge) and minutely velutinous-papillate on the haft and lower half of the adaxial surface, a remarkable character that contrasts with the smooth falls of *Portiodora*. The species was collected in an unspecified location in “Fokien” (presently Fujian Province) by C. de Grijs, to whom the epithet is dedicated. He also added “Inter *I. ensatam* et *I. ruthenicam*, huic tamen propior” [between *I. ensata* and *I. ruthenica*, though closer to the latter], and listed the similarities and dissimilarities of *I. grijsii* versus both putative relatives. Notwithstanding the absence of data pertaining to seeds and fruits in the protologue of *I. grijsii*, Hance [19] had previously delineated those features from the specimens in his herbarium that he called “*I. oxypetala* Bge.”, which Maximowicz [54] referenced in the protologue and used as type material for his new species. The description of Hance outlines capsules that are pergameneous, fusiform, long-beaked, equidistantly 6-ribbed and reticulate-nerved between ribs; and seeds that are globose, polygonal and devoid of fleshy appendages. Those characteristics do not align with *Portiodora speculatrix*. Unfortunately, such data were not reported for *I. cavaleriei* (Figure 8b), which would facilitate refining its morphological relationships with other groups of irises. The latter species was described by Léveillé [55] from plants found by J. Cavalerie in “Kouy-Tchéou: Kouy-yang, bord de fleuve” [Guizhou: Guiyang, river edge], and Léveillé also reported the presence of *I. grijsii* in “Fukien, Ngan-Hoei” [Fujian and Anhui Provinces] in the original publication. Although both *I. grijsii* and *I. cavaleriei* appear to have certain affinities with the species of *Eremiris* or perhaps *Joniris*, transference to any of those genera is not favoured.

Related to this subject, Su [56] recently published a remarkable species from Fujian Province, *Iris fujianensis* X.X.Su (nom. inval., holotype not cited), which shares morphology and distribution with *I. grijsii* and *P. speculatrix*. Important differences exist among these three species (mostly concerning the characteristics and sizes of leaves, flowers and fruits and the arrangement of the inner floral segments), though some others such as the general habit, rhizome growth and the putative entire triangular stigmatic lip (not mentioned in the protologue of the two former) suggest a presumed closeness among them. More importantly, *I. grijsii* and *I. fujianensis* bear several short leaves at the base of the scape and show outer floral pieces similar in morphology and ornamentation, which exhibit blades with a remarkable velutinous surface that has not been documented in *Portiodora* and most members of the *Xiphion* s.l. clade. Similarly, *I. grijsii* produces oblong capsules with a long apical beak, and its ripe seeds are angulose and apparently lack appendages. However, the protologue of *I. fujianensis* [56] describes a seed structure resembling a visible peduncle that might correspond to the funicle, but it is not referred to as a fleshy appendage after ripening (perhaps evanescing with maturation). More precise data are needed about this crucial character, which also would connect it to *Portiodora*. Furthermore, the innermost floral pieces spread outwards in *I. fujianensis* but are erect to subpatent in *I. grijsii*. Further detailed observations are required to clarify the morphological connections of *I. fujianensis* to other Asian irises. Despite the similarities that can be drawn with *Eremiris* or potentially *Joniris* (as for *I. grijsii*) or in some respect to *Portiodora*, the species possesses a unique morphology that enable it to be distinguished from other irises [54], i.e., leaves longer and broader (20–80 × 1.4–2.6 cm), flowers solitary and borne on shorter pedicels (1–2 cm long) and with larger pieces (7–8 × 2.0–2.5 cm) spreading outwards. Those divergences may perhaps require further description of an unedited generic lineage.

In any case, until the required full morphological details and DNA sequence data are available for *I. grijsii*, *I. cavaleriei* and *I. fujianensis*, their phylogenetic relationships and generic placement in the “*Iris*-flower clade” [sensu 14] will remain unresolved. At the moment, based on the available morphological evidence, we provisionally exclude them from *Portiodora*, but this hypothesis requires further testing.

### 4.6. Some Reflections on the Current Vision on Iris-Segregated Genera

Iridology, like other disciplines dealing with economically important plants, is subject to a great immobility regarding the application and use of scientific names. Researchers are reluctant to change their conventional concept of a very broadly circumscribed genus *Iris* amalgamating widely variable infrageneric groups of irises [17], which apparently bring nomenclatural stability [57] by avoiding the use of “too many genera”. Such broad treatments are perhaps based mostly on tradition rather than on integrative taxonomic data (e.g., morphology, karyology, phytochemistry, molecular phylogenetics, biogeography, etc.) that can support more analytical treatments in the “*Iris*-flower clade” (see recent discussion by Crespo et al. [18]).

Traditionally, horticulturists and specifically iridologists have utilised “working names” for more than 150 years to denominate each of the clades and lineages recovered in recent molecular work, a fact that does not imply a loss of nomenclatural stability or generate any confusion. In the recent multi-generic treatment of the irises [13], most of those traditional working names were recognised at the genus rank, which paralleled other accepted analytical treatments in horticulturally important iridaceous groups such as Crocoideae (see [58]), or other families of bulbous plants such as Hyacinthaceae ≡ Asparagaceae subfam. Scilloideae (see [59,60,61,62,63]).

Popular nomenclature does not necessarily need to align with scientific nomenclatural changes. Obviously, common people will invariably refer to daisies, poppies, aloes and irises by these same names, irrespective of their scientific classification within a single genus or multiple genera [18]. Paradoxically, it is interesting to note that the adoption of new treatments by taxonomists is often dependent on the economic importance of the concerned groups, with an increasing reluctance to apply nomenclatural changes to economically relevant aggregates. This is a common assumption, despite the apparent contradiction of applying divergent criteria depending on the extra-scientific impact of the groups concerned (for further details see [18]).

New taxonomic rearrangements, predominantly promoted in the wake of molecular phylogenies, have been documented over the past two decades. The majority of these rearrangements result in a significant simplification of the taxonomy of the relevant aggregates, often leading to more homogeneous segregate genera that are more manageable [14,61,62,63]. They are typically smaller and well defined on the basis of morphological, molecular and biogeographical characteristics. In this context, the authors advocate the implementation of these homogeneous taxonomic criteria across all *Iridaceae*, for which the multi-generic arrangement of the “*Iris*-flower clade” represents a significant step forward.

### 4.7. Implications on Biodiversity Conservation

The use of broadly defined genera clearly biases the way in which researchers perceive both morphological and taxonomic diversity within a given group, such as the family *Iridaceae*. This conceptual narrowing may have serious negative consequences for conservation practices. For example, the potential extinction of *I. speculatrix* would not result in the disappearance of the entire genus *Iris*. Therefore, it would likely be regarded by many as just an unfortunate loss of one of its local forms, externally like the one growing in a domestic garden bed. However, it is unsurprising that the extinction of an entire genus of flowering plants (*Portiodora*), which had unique morphology and genetics, would be regarded as a far more tragic event. Such psychological differences in perception cannot be ignored or underestimated, as they will inevitably influence the intensity of conservation efforts directed toward objects facing potential extinction. The example of the so-called “Louisiana irises” [6] clearly demonstrates that in situations where conservation efforts conflict seriously with the economic interests of the State and corporations, prioritising broad traditional taxonomy over detailed taxonomic elaboration can have disastrous consequences [6].

Without delving into the much broader topic of reconciling phylogenetic and Linnaean (rank-based) classifications, it is still worth emphasising that, bearing in mind the obtained phylogenetic outcomes (Figure 6, Figure 7 and Figures S1–S3), the acceptance of the newly described genus *Portiodora* as a section or subgenus does not appear to be intuitively convincing; especially when genera such as *Chamaeiris*, *Juno* or *Iridodictyum* are simultaneously accepted at the same ranks.

## 5. Conclusions

Within an integrative taxonomic framework, the combination of morphological, molecular, karyological and geographical data provides sufficient evidence to place *Iris speculatrix* in the new genus *Portiodora*, which completes the multi-generic treatment adopted by Crespo et al. [13] for the “*Iris*-flower clade”. The morphological features of this enigmatic and pleasing evergreen iris appear to link it to multiple distinct groups. However, the unique morphology it displays, in conjunction with its outstanding karyological peculiarities and its isolated phylogenetic position within the “*Iris*-flower clade” support the generic rank for *I. speculatrix*.

The acceptance of *Portiodora* as an autonomous genus is in line with the rationale presented by Mavrodiev et al. [14] and Crespo et al. [13,18]. The stability of any proposed new arrangement depends on its integrative nature, i.e., the successful combination of phylogenetic, morphological and biogeographical information, as well as its comprehensiveness, so that it does not exhibit taxonomic biases or adhere to regional botanical practices [13,60,61,62,63]. The pure comparative approach to molecular data (three-taxon statement analysis) is a practical methodology for addressing taxonomic questions, since it eschews assumptions about the history of chloroplast loci transformation [64], and may therefore be applicable to similar studies. In that context, admitting monotypic or small genera such as *Portiodora* (as for other lineages of the “*Iris*-flower clade”) is not a drawback. Conversely, and irrespective of their size, these smaller groups facilitate and even render more realistic the understanding of the diversity, complexity and relationships of the irises, and have an important impact on their conservation. In this sense, the present study provides a framework for a future protective strategy for the entire “*Iris*-flower clade”. The accurate resolution of the taxonomy of cryptic biodiversity, such as that exhibited by *Portiodora*, is likely to be essential for the successful conservation of this group.

## Figures and Tables

**Table 1:** Main morphological features of *Portiodora* and related rhizomatous genera of the “*Iris*-flores clade” with which it was related.

Characteristics	*Portiodora*	*Zhaoanthus*	*Eremiris*	*Joniris*	*Cryptobasis*	*Syrianthus*	*Chamaeiris*
Rhizome	Stout, nodose, creeping	Slender, wiry, stoloniferous, many-branched	Thick, compact, creeping, branched	Slender, many-branched, creeping	Slender, nodose, vertical	Stout, nodose, nearly vertical	Stout, nodose, creeping
Stems	Long, visible	Long, visible, simple	Long, visible, simple	Short, slender, simple	Hidden among leaf remains	Long, visible, simple	Long, visible, simple to few-branched
Keel of spathe valves	Absent	Present	Present	Absent	Present	Present	Present
Perigone tube shape and length	Cylindric, 5–8 mm long	Cylindric, 1–70 mm long	Cup-like, 1–3 mm long	Cylindric, 5–15 mm long	Cylindric, 40–120 mm long, scapiform	Cylindric, up to 20 mm long	Cup-like,2–27 mm long
Outline of falls	Obovate-spatulate	Spatulate	Oblanceolate	Broadly oblanceolate	Panduriform to spatulate	Oblanceolate to panduriform	Panduriform or subspatulate
Fall position	Erect-patent	Erect-patent	Erect-patent	Erect-patent	Erect-patent to patent	Erect to erect-patent	Patent
Style branches length	Slightly shorter than falls	Half the length of falls	About half the length of falls	About half the length of falls	Slightly shorter than falls	Slightly shorter than falls	About half the length of falls
Crest of falls	Low sinuous near the middle, no lateral outgrowths	Low, wavy, with lateral outgrowths	Absent	Absent	Absent	Absent	Absent
Stigmatic lip	Entire, triangular-obtuse	Entire, oblong to triangular	Triangular, long acuminate	Triangular, apiculate	Bilobed, with rounded lobes	Bilobed, with rounded lobes	Bifid, with acute lobes
Capsule shape	Oblong-fusiform, subtrigonous	Ellipsoid to subglobose, trigonous	Oblong-cylindrical to fusiform, trigonous,	Globose to obovoid, trigonous	Ovoid to cylindric, not trigonous	Cylindricalellipsoid, trigonous	Ovate-lanceolate to oblong, not trigonous
Capsule position regarding stem and spathes	Patent, long exerted	Erect, often exerted	Erect, often exerted	Erect, hidden into spathes	Erect, often exerted	Erect, often exerted	Erect, often exerted
Capsule ribs	3, prominent	3, prominent	6, weak	6, weak	6, prominent	6, weak	6, prominent
Capsule beak	Present	Present	Present	Absent	Present	Absent	Present
Capsule dehiscence	From apex to base	From apex to about middle	From apex to about middle	From apex to about middle	From apex to about middle	From apex to about middle	From apex to about middle
Capsule valves	Curled backwards	Erect to erect-patent	Erect to erect-patent	Curled backwards	Erect to erect-patent	Erect to erect-patent	Erect to curled backwards
Seed morphology	Globose-angulose	Globose compressed	Pyriform, subapiculate	Globose to pyriform	Angulose to subcubic	Globose, necked	Globose to subcubic
Seed appendages	Aril, withering as a wing	Fleshy raphe	Absent	Fleshy raphe, vanishing	Absent	Absent	Absent
Seed testa	Rugulose	Slightly wrinkled.	Smooth, shiny	Smooth	Wrinkled on faces, smooth on back	Tuberculate, hard	Fleshy and smooth or loose-papery

**Table 2:** Main karyotype features of *Portiodora* and karyologically related rhizomatous genera of the “*Iris*-flower clade”.

Genus	Chromosomal Size Structure	Satellited Chromosomes	Secondary Constrictions	Telocentric and/or Subtelocentric Chromosomes
*Portiodora*	Homogeneous	1 pair	Absent	Absent
*Chamaeiris*	Heterogeneous	0	Absent	Absent
*Zhaoanthus*	Heterogeneous	0	Absent	Present
*Rodionenkoa*	Heterogeneous	1 pair	Absent	Present
*Phaeiris*	Heterogeneous	2–3 pairs	Present	Present
*Joniris*	Subhomogeneous	1 pair	Absent	Absent
*Eremiris*	Subhomogeneous	1 pair	Absent	Present
*Lophiris*	Heterogeneous	1–2 pairs	Absent	Absent
*Evansia*	Heterogeneous	(0–)2 pairs	Absent/present	Present
*Tectiris*	Heterogeneous	(0–)2 pairs	Absent/present	Absent
*Iris*	Heterogeneous	0–1 pair	Absent/present	Present

## Data Availability

The DNA sequences referred to in the present research are available at GenBank (https://www.ncbi.nlm.nih.gov/genbank/, accessed on 30 September 2025).

## Supplementary Materials

The following supporting information can be downloaded at: https://www.mdpi.com/article/10.3390/biology14121767/s1, Figure S1: Hierarchy patterns of score 23 representing the Robinson–Foulds median consensus of eight cladograms (Table S1), the results of 3TA analyses of *Iris* s.l. and outgroups supermatrix (202 terminals); Figure S2: Cladogram representing the most probable maximum likelihood (ML) tree (−ln likelihood = −48,909.1674), inferred using IQ-TREE from a plastid supermatrix of *Iris* s.l. and outgroup taxa (206 terminals). The best-fit model, TVM+F+I+G4, was automatically selected by IQ-TREE according to the Schwarz (Bayesian) Information Criterion. The aLRT support values (see Mavrodiev et al. [14] for reference) were rounded to the nearest whole number and shown above or below branches when equal to 75% or higher; Figure S3: The simplified version of Figure 5 from the main text; Table S1. Summary of input data for final 3TA cladogram construction (Figure 6), following Mavrodiev et al. [14]. Each row corresponds to a separate 3TA analysis and represents a summary that includes the number of ingroup terminals, the number of three-taxon statements, the number of resulting hierarchies (trees), tree length, tree retention index (RI), and the Robinson–Foulds (RF) median consensus (MC) score (distance). See the main text for references.

## Appendix A. Identification Key

The dichotomous key for identification of genera in the “*Iris*-flower clade” that was previously published by Crespo et al. [13] is updated below to include the new genus *Portiodora*.

1. Perigone segments all equal, free; style branches filiform, non-petaloid; capsule valves split down to base; seeds fleshy, blackish, globose, long persisting on the fruit axis (resembling a blackberry).......................................................***Belamcanda***

− Perigone segments unequal (falls and standards), fused in a tube or rarely free; style branches flattened, petaloid; capsule and seeds with other characteristics.........2

2. Leaves bifacial (dorsiventrally flattened), usually canaliculate, sometimes polygonal in cross section.....................................................................................................3

− Leaves isobilateral (laterally flattened), usually equitant........................................7

3. Roots fleshy, swollen, persistent; standards patent to reflexed; falls obovate, usually with a prominent central crest..............................................................................***Juno***

− Roots fibrous, usually not fleshy neither swollen, deciduous; standards erect; falls panduriform, not crested or with a weak, inconspicuous crest......................4

4. Basal leaves quadrangular to octagonal in cross section; seeds appendiculate........5

− Basal leaves semicircular to circular in cross section, sometimes with prominent ribs or keel; seeds not appendiculate.........................................................................6

5. Rootstock a bulb, with outer tunics reticulate, sometimes enclosing several offsets; standards conspicuous, longer than half the style branches length; ovary and capsule trilocular..................................................................................................***Iridodictyum***

− Rootstock with 2–4 oblong tubercles, ±digitate; standards inconspicuous, shorter than half the style branches length; ovary and capsule unilocular....***Hermodactylus***

6. Bulb outer tunics reticulate. Leaves prominently ribbed on the outer side, sometimes keeled; stem inconspicuous, underground; stigma entire........................***Alatavia***

− Bulb outer tunics membranous to somewhat fibrous, not reticulate. Leaves without prominent ribs on the outer side, not keeled; stem conspicuous, aerial; stigma bifid, usually with acute lobes.................................................................................***Xiphion***

7. Flowering stems with marked dichotomous branching pattern; flowers short-lived, shrivelling spirally and falling off just below the ovary; pedicels persistent, stiff after flower abscission; seeds winged.............................................***Pardanthopsis***

− Flowering stems simple or without evident dichotomous branching pattern; flowers not shrivelling spirally, falling off just above the ovary; pedicels usually not stiff after flower abscission; seeds mostly unwinged, sometimes with fleshy cover or fleshy appendages......................................................................................................8

8. Both perigone whorls usually similar in size and shape; beard (a very conspicuous central row of thick pluricellular, multiseriate, clavate hairs) present on falls and sometimes also on standards; fruit opening by clefts, the valves remaining fused apically for a long time after dehiscence........................................................***Iris***

− Both perigone whorls usually strongly differing in size and/or shape; beard absent, or falls sometimes crested, pubescent or with slender unicellular hairs; fruit valves splitting completely from the apex up to about the middle..................................9

9. Falls markedly crested, with 1–3 fringed, irregularly dissected or wavy rows......10

− Falls uncrested or sometimes with an almost entire low crest or central ridge......14

10. Rhizome slender, long, heterogeneous, with cord-like branches at apex, swollen at nodes; leaves herbaceous, without prominent ribs; falls with crest of three fringed rows; seeds with a long, coiled appendage...............................................***Lophiris***

− Rhizome lacking cord-like branches at the apex; leaves ± coriaceous, with 1–5 prominent ribs; falls with crest of a single fringed row, sometimes with lateral outgrowths; seeds lacking coiled appendages......................................................11

11. Fall crest mostly wavy; stigma oblong to triangular, entire; capsule beaked............................................................................................................***Zhaoanthus***

− Fall crest fringed or dissected; stigma bilobed, with broad lobes; capsule ± pointed, not beaked.................................................................................................................12

12. Rootstock a small rhizome, with many swollen tuberous roots; perigone pieces entire or weakly erose-undulate on margins; fall crest not flanked with lateral pairs of outgrowths near the base............................................................***Junopsis***

− Rootstock a thick rhizome, usually nodose and sometimes stoloniferous, lacking swollen, tuberous roots; perigone pieces markedly undulate-erose on margins; fall crest flanked with 2–3 lateral pairs of outgrowths near the base................13

13. Haft of the standards inconspicuous, short, nearly flat; capsule subcoriaceous; seeds brownish, matte, irregular in shape, angular, ±flattened..............***Evansia***

− Haft of the standards conspicuous, long, canaliculate; capsule papery; seeds blackish, glossy, globose-pyriform, apiculate......................................................***Tectiris***

14. Stigma triangular or linguiform, entire or with crenate margins....................15

− Stigma bilobed to bifid, sometimes with denticulate lobes..................................21

15. Style crests long triangular-lanceolate; capsule with a conspicuous long beak.....16

− Style crests broadly triangular to roundish; capsule unbeaked or shortly beaked...18

16. Nectar drops at the base of falls; stamen filaments fused into a tube; seeds covered with glistening glands, lacking fleshy appendages........................***Siphonostylis***

− Nectar drops lacking at the base of falls; stamen filaments not fused into a tube; seeds not glandulous, with a fleshy wing-like appendage..................................17

17. Leaves not glossy, lacking prominent ribs; stem short, hidden under spathes; perigone tube 25−65 mm long; falls with a central band of minute, velvety pubescence; capsule erect, short-beaked..........................................................***Gattenhofia***

− Leaves glossy, prominently ribbed; stem elongated, clearly visible; perigone tube up to 8 mm long; falls smooth, not pubescent; capsule patent, long-beaked..***Portiodora***

18. Rhizome slender, long, heterogeneous, with cord-like branches at apex, swollen at nodes; leaves without prominent ribs; testa surface pitted..............***Rodionenkoa***

− Rhizome lacking cord-like branches at apex; leaves usually with prominent ribs; testa surface not pitted...........................................................................................19

19. Leaves firm, coriaceous, up to 40 mm wide; seeds angular, discoid or semidiscoid, usually corky; testa surface smooth or papillate....................................***Limniris***

− Leaves slender, up to 15 mm wide; seeds globose to pyriform, sometimes compressed, not corky; testa surface ± smooth, sometimes glossy..........................20

20. Rhizome slender, up to 5 mm in diameter; perigone tube up to 15 mm long; capsule globose to ovoid, 10–15 mm long; seeds with a fleshy appendage vanishing on drying......................................................................................................***Joniris***

− Rhizome stout, up to 10 mm in diameter; perigone tube up to 3 mm long; capsule oblong-cylindrical to fusiform, 20–80 mm long; seeds lacking fleshy appendages..........................................................................................................***Eremiris***

21. Rhizome almost vertical; style crests lanceolate-triangular...........................22

− Rhizome long, creeping; style crests broadly triangular, subquadrate (rarely narrowly triangular)..............................................................................................24

22. Rootstock with needle-like bristles at the apex; rhizome tough, nearly vertical, with compact, bulbiform, swollen leaf bases; shoots extra– or intravaginal; spathe valves 2; perigone tube up to 2 cm long; capsule unbeaked; seeds tuberculate, necked...................................................................................................***Syrianthus***

− Rootstock covered with brownish remains of leaf sheaths, not spiny; leaves not bulbiform at the base; plants forming dense tussocks, with all shoots intravaginal; spathe valves 3–4; perigone tube 4–12 cm long; capsule beaked; seeds wrinkled, unnecked........................................................................................23

23. Stems absent or hidden among leaf remains; perigone tube scapiform; crest triangular-lanceolate to oblong-lanceolate, irregularly fringed on margins; capsule 6-ribbed, not reticulate-nerved...........................................***Cryptobasis***

− Stems distinct; perigone tube not scapiform; crests narrowly triangular-lanceolate to linear, with slightly crenate to almost entire margins; capsule, trigonous, reticulate-nerved..................................................................................***Sclerosiphon***

24. Haft of falls patent, without a central ridge; capsule conspicuously beaked; seed testa fleshy and smooth or loose-papery with irregularly ridged surface........***Chamaeiris***

− Hafts of falls erect, with a central ridge; capsule with rounded to acute apex, rarely with a short beak; seed testa corky or hard, with wrinkled or smooth surface.....25

25. Falls glabrous; capsule rounded in cross section; seeds nearly globular, with testa wrinkled...........................................................................................***Dielsiris***

− Falls pubescent on or aside ridge; capsule ± hexagonal in cross section; seeds semidiscoid to discoid, flattened, ±angular, with testa corky, ±smooth.....***Phaeiris***
