# Supplementary material for: Watching the South China Sea—Portiodora (Iridaceae), a New Genus for Iris speculatrix Based on Comprehensive Evidence: The Contribution of Taxonomic Resolution to Biodiversity Conservation
Source: Biology (Basel). 2025 Dec 11;14(12):1767. doi: 10.3390/biology14121767 (PMC12730399; doi:10.3390/biology14121767)
Supplement: Supplementary file 1 [file biology-14-01767-s001.zip › biology-3988196-supplementary.pdf]

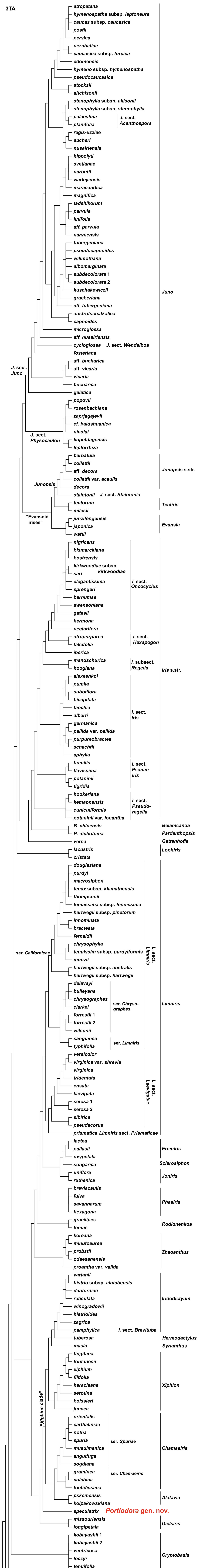

Figure S1. Hierarchy patterns of score 23 representing the Robinson-Foulds median consensus of eight cladograms (Table S1), the results of 3TA analyses of *Iris* s.l. and outgroups supermatrix (202 terminals).

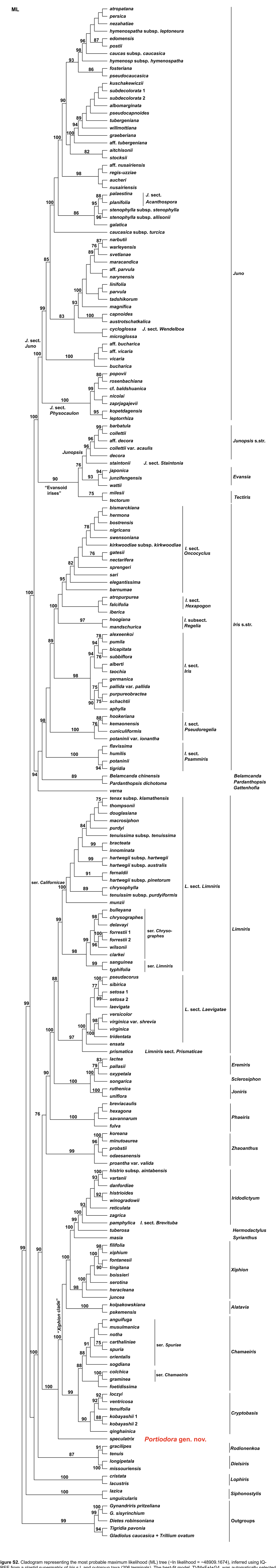

Figure S2. Cladogram representing the most probable maximum likelihood (ML) tree (−ln likelihood = −48909.1674), inferred using IQ-TREE from a plastid supermatrix of *Iris* s.l. and outgroup taxa (206 terminals). The best-fit model, TVM+F+I+G4, was automatically selected by IQ-TREE according to the Schwarz (Bayesian) Information Criterion. The aLRT support values (see Mavrodiev et al. [14] for reference) were rounded to the nearest whole number and shown above or below branches when equal to 75% or higher.

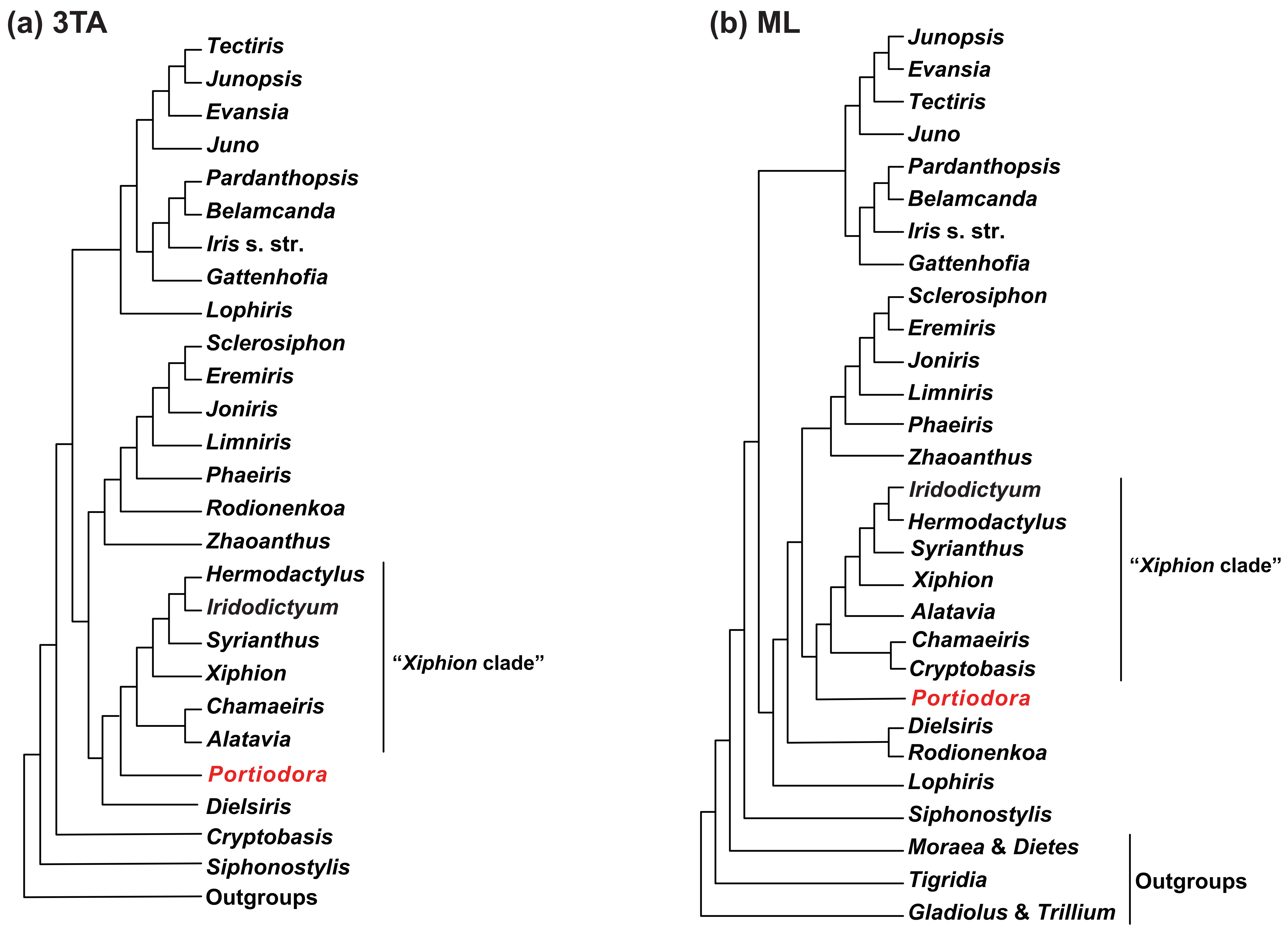

Figure S3. The simplified versions of Figures 6 (a) and 7 (b) from the main text.

Table S1. Summary of input data for final 3TA cladogram construction (Figure 6), following Mavrodiev et al. [14]. Each row corresponds to a separate 3TA analysis and represents a summary that includes the number of ingroup terminals, the number of three-taxon statements, the number of resulting hierarchies (trees), tree length, tree retention index (RI), and the Robinson–Foulds (RF) median consensus (MC) score (distance). See the main text for references.

	The name of the 3TA analysis	Number of ingroup terminals	Number of three-taxon statements	Number of 3TA hierarchies (trees)	3TA Tree length	RI	Score (distance) of RF MC
1	“Summary”	85	2279872	9	2956642	0.7032	24
2	*Juno*	58	377503	100	451182	0.8048	1410
3	*Evansia*	10	1159	1	1240	0.9301	n\a
4	*Iris* s. str.	37	63697	90	68959	0.9174	1188
5	*Limniris - Phaeiris*	45	312591	780	368895	0.8199	684
6	*Eremiris*	7	611	1	631	0.9673	n\a
7	*Cryptobasis*	7	80	8	97	0.7875	22
8	"*Xiphion* clade"	32	155857	20	189660	0.7831	72
